# Proceedings of Fox River Valley Medical Society

**Published:** 1865-08

**Authors:** D. W. Young, Jos. Tefft

**Affiliations:** Sec.; Pres.


### PROCEEDINGS OF THE FOX RIVER VALLEY MEDICAL ASSOCIATION.

The Association met in the Common Council room in the city of Elgin, on Monday afternoon, July 3d, 1865. The president, Dr. Tefft, in the chair.

Present, Drs. Tefft, Howell, Burdick, Woodworth, Tyler, Jassoy, Bartels, Hawley, Eddy, Reed, and Young. Minutes of last meeting read and approved. Dr. Howell proposed Dr. John Jassoy for membership.

On motion of Dr. Young, Dr. Jassoy was elected, when he came forward, paid the admission fee, signed the constitution, and duly became a member of this Association.

Dr. Hawley announced that the Chicago *Republican* refused to publish *criminal* quack advertisements, and that the Ohio State Medical Association had passed resolutions commending its course, and moved that a committee be appointed by the chair to draft resolutions expressive of the sense of this Association. The President appointed Drs. Hawley, Howell, and Woodworth such committee.

Dr. Tefft read an interesting paper giving an account of a case of cancer of the stomach that had occurred in his practice recently. Dr. Burdick reported a case of malignant tumor of the jaw that was successfully removed by an operation. Dr. Young reported an interesting case of abscess of the lungs in a child. The child recovered after a protracted illness.

Dr. Tefft presented a patient with inflammation and swelling of the inferior extremities of long standing. Quite a discussion ensued as to the pathology of the case, which was participated in by Drs. Howell, Jassoy, Tefft, Hawley, Tyler and Young.

The committee on resolutions reported as follows:

*Whereas*, the admission by the public press of advertisements of an immoral character, inviting the commission of crime by producing abortion and preventing pregnancy for a consideration, in the shape of pills, potions, drops, etc., is terribly demoralizing and destructive of health and life, we therefore hail with pleasure the promise of the Chicago *Republican* that they will not admit advertisements of this character into their paper; there ore—

*Resolved*, that the moral integrity and noble stand of its proprietors in excluding this class of advertisements from its columns merits our highest approbation, and we hereby pledge ourselves to take and recommend only such papers as best conform with the principles of morality on this subject, believing that we, as guardians of the public health and conservators of public morals, can not too strongly reprobate the publication in religious papers of advertisements (for money,) the object of which is clearly the prevention of pregnancy, or the commission of child murder, to commit crime, pander to lust, and that too frequently at the expense of the mothers, as well as their offspring.

*Resolved*, that the wide-spread influence of a certain looseness of sentiment on this subject should be a matter of great alarm to the philanthropic physician, as well as the parent, and that every public print that will pander to this species of crime, is wickedly suicidal of the best interests of our race, and unworthy to be received into family circles or the patronage of honest men.

*Resolved*, that we deem those druggists who advertise and sell such preparations as *particeps criminis ;* we hereby earnestly recommend them to discard them from their stocks and refuse to furnish them.

The report was accepted and adopted, and the Secretary instructed to publish them in the Chicago Medical Journals, *Republican* and Aurora *Beacon.*

Dr. Burdick moved that a vote of thanks be tendered the proprietors of the Aurora *Beacon* for their generosity and promptness in publishing the proceedings of our Association. The resolution was adopted unanimously.

On motion of Dr. Burdick, the Association adjourned to meet in the common council room in the city of Aurora, on the 1st Monday of October next, at 1 o'clock, P. M.

D. W. Young, M. D., Sec. Jos. Tefft, M. D., Pres.

